# Functional Characterization of Human Induced Pluripotent Stem Cell-Derived Endothelial Cells

**DOI:** 10.3390/ijms23158507

**Published:** 2022-07-31

**Authors:** Xuehui Fan, Lukas Cyganek, Katja Nitschke, Stefanie Uhlig, Philipp Nuhn, Karen Bieback, Daniel Duerschmied, Ibrahim El-Battrawy, Xiaobo Zhou, Ibrahim Akin

**Affiliations:** 1 Department of Cardiology, Angiology, Hemostaseology and Medical Intensive Care, Medical Faculty Mannheim, University Medical Centre Mannheim (UMM), Heidelberg University, 68167 Mannheim, Germany; xuehui.fan@medma.uni-heidelberg.de (X.F.); daniel.duerschmied@medma.uni-heidelberg.de (D.D.); ibrahim.elbattrawy2006@gmail.com (I.E.-B.); ibrahim.akin@umm.de (I.A.); 2 Key Laboratory of Medical Electrophysiology of Ministry of Education, Medical Electrophysiological Key Laboratory of Sichuan Province, Collaborative Innovation Center for Prevention and Treatment of Cardiovascular Disease, Institute of Cardiovascular Research, Southwest Medical University, Luzhou 646000, China; 3 European Center for AngioScience (ECAS) and German Center for Cardiovascular Research (DZHK) Partner Site Heidelberg/Mannheim, 68167 Mannheim, Germany; 4 DZHK (German Center for Cardiovascular Research), Partner Site, 37075 Göttingen, Germany; lukas.cyganek@gwdg.de; 5 Stem Cell Unit, Clinic for Cardiology and Pneumology, University Medical Center Göttingen, 37075 Göttingen, Germany; 6 Department of Urology and Urosurgery, Medical Faculty Mannheim, Heidelberg University, 68167 Mannheim, Germany; katja.nitschke@umm.de (K.N.); philipp.nuhn@medma.uni-heidelberg.de (P.N.); 7 Flow Core Mannheim Medical Faculty Mannheim, Heidelberg University, 68167 Mannheim, Germany; stefanie.uhlig@medma.uni-heidelberg.de (S.U.); karen.bieback@medma.uni-heidelberg.de (K.B.); 8 Institute of Transfusion Medicine and Immunology, Medical Faculty Mannheim, Heidelberg University, 68167 Mannheim, Germany; 9 Bergmannsheil Bochum, Medical Clinic II, Department of Cardiology and Angiology, Ruhr University, 44789 Bochum, Germany

**Keywords:** human-induced pluripotent stem-derived endothelial cells, human cardiac microvascular endothelial cells, ion channel, endotheline-1, nitric oxide, exosome

## Abstract

Endothelial cells derived from human induced pluripotent stem cells (hiPSC-ECs) provide a new opportunity for mechanistic research on vascular regeneration and drug screening. However, functions of hiPSC-ECs still need to be characterized. The objective of this study was to investigate electrophysiological and functional properties of hiPSC-ECs compared with primary human cardiac microvascular endothelial cells (HCMECs), mainly focusing on ion channels and membrane receptor signaling, as well as specific cell functions. HiPSC-ECs were derived from hiPS cells that were generated from human skin fibroblasts of three independent healthy donors. Phenotypic and functional comparison to HCMECs was performed by flow cytometry, immunofluorescence staining, quantitative reverse-transcription polymerase chain reaction (qPCR), enzyme-linked immunosorbent assay (ELISA), tube formation, LDL uptake, exosome release assays and, importantly, patch clamp techniques. HiPSC-ECs were successfully generated from hiPS cells and were identified by endothelial markers. The mRNA levels of KCNN2, KCNN4, KCNMA1, TRPV2, and SLC8A1 in hiPSC-ECs were significantly higher than HCMECs. AT1 receptor mRNA level in hiPSC-ECs was higher than in HCMECs. AT2 receptor mRNA level was the highest among all receptors. Adrenoceptor ADRA2 expression in hiPSC-ECs was lower than in HCMECs, while ADRA1, ADRB1, ADRB2, and G-protein GNA11 and Gai expression were similar in both cell types. The expression level of muscarinic and dopamine receptors CHRM3, DRD2, DRD3, and DRD4 in hiPSC-ECs were significantly lower than in HCMECs. The functional characteristics of endothelial cells, such as tube formation and LDL uptake assay, were not statistically different between hiPSC-ECs and HCMECs. Phenylephrine similarly increased the release of the vasoconstrictor endothelin-1 (ET-1) in hiPSC-ECs and HCMECs. Acetylcholine also similarly increased nitric oxide generation in hiPSC-ECs and HCMECs. The resting potentials (RPs), I_SK1–3_, I_SK4_ and I_K1_ were similar in hiPSC-ECs and HCMECs. I_BK_ was larger and I_KATP_ was smaller in hiPSC-ECs. In addition, we also noted a higher expression level of exosomes marker CD81 in hiPSC-ECs and a higher expression of CD9 and CD63 in HCMECs. However, the numbers of exosomes extracted from both types of cells did not differ significantly. The study demonstrates that hiPSC-ECs are similar to native endothelial cells in ion channel function and membrane receptor-coupled signaling and physiological cell functions, although some differences exist. This information may be helpful for research using hiPSC-ECs.

## 1. Introduction

Cardiovascular diseases (CVD) remain one of the leading causes of mortality in the world, and is often related to endothelial dysfunction [1]. Endothelial cells (ECs) are arranged in the lumen of all blood vessels and play a critical role in regulating blood vessel permeability, contraction/relaxation, angiogenesis, tissue regeneration, and homeostasis. The emergence of human embryonic stem cells (hESCs) and human induced pluripotent stem cells (hiPSCs), derived from various somatic cell origins, pave the way to generate patient-specific ECs, which may be used as a powerful in vitro disease model, can be used for drug screening, transplantation, regenerative and translational medicine, and can provide an unlimited source of autologous cells [1,2,3]. Multiple protocols of differentiating hiPSCs into endothelial cells have been established. However, an efficient, high-purity and cost-effective method for generating human induced pluripotent stem cell (hiPSC)-derived endothelial cell (hiPSC-EC) still needs to be established.

The protocol of obtaining hiPSC-EC consists of three key steps: mesoderm induction, endothelial specification, and EC purification [1,4,5,6,7]. The hiPSC-ECs may not be as mature and stable as adult endothelial cells. Currently, due to low yield and significant heterogeneity, researchers have been exploring new signaling pathways, or developing new culture conditions or cell reprogramming to improve EC differentiation efficiency and purity or to improve their function. For example, blockade of the sterol regulatory element binding protein 1 (SREBP1), inhibition of the MEK/ERK pathway and the Notch signaling pathway, inhibition of miR-495, activation of both p38 MAPK and ERK1/2 signaling pathways, and the application of a 3D porous fibrin scaffold increased the ability of both hiPSC differentiation and EC angiogenesis [8,9,10,11,12,13]. Endothelial cell-specific molecules 1 (ESM1) is one of the most expressed genes in hiPSC-ECs. It regulates connexin 40 (CX40) and eNOS, further affecting EC enrichment and function [14]. Xenogeneic-free hiPSC-ECs (XF-hiPSC-ECs) that are similar to human primary ECs in marker expression and function, can further promote the clinical application of tissue-engineered vascular grafts (TEVGs) therapy [15]. Mitochondrial maturation is essential to maintain hiPSC-EC functionality [16].

Patient-specific hiPSC-ECs may be widely used to replace damaged or dysfunctional ECs in the systemic vasculature and provide an excellent platform for studying vascular development and CVD. HiPSC-ECs promoted the perfusion of ischemic tissue in a murine model of peripheral arterial disease [17]. In addition, hiPSC-ECs have been widely used in myocardial infarction [18], or ischemia diseases [19,20,21]. Exosomes secreted by hiPSC-derived cardiomyocytes (hiPSC-CMs), endothelial cells, and smooth muscle cells improved myocardial recovery [18]. Exosomes from hiPSC-CMs promoted tube formation, migration, and proliferation of hiPSC-ECs [22]. Exosomes secreted from glomerular endothelial cells treated with high glucose trigger the epithelial-mesenchymal transition and dysfunction of podocytes [23]. However, the comparison of functional characteristics of exosomes secreted by hiPSC-ECs and native endothelial cells has not been reported yet.

Endothelial dysfunction can result from the changed expression profile of ion channels and abnormal electrical property. The alternation of ion channel subunit expression in human coronary artery ECs is related to flow adaptation [24]. Inactivation of small/intermediate conductance of calcium-activated potassium channels (SKCa/IKCa) in endothelial cells contributes to endothelial dysfunction [25,26,27,28,29]. Large conductance, Ca^2+^-activated K^+^ (BK) activation with NS1619 decreased LPS-induced CCL-2 secretion in human pulmonary microvascular endothelial cells [30]. However, the electrophysiological properties of ion channels in hiPSC-ECs have not yet been reported. Furthermore, studies on functional properties of hiPSC-ECs are sparse. Therefore, we investigated electrophysiological and functional characterization of hiPSC-ECs and compared them with native human cardiac microvascular endothelial cells (HCMECs), to clarify the similarities and differences between hiPSC-derived ECs and HCMECs, which may provide useful information for studies using hiPSC-ECs.

## 2. Results

### 2.1. Characterizations of hiPSC-ECs

To induce the differentiation of human iPSC to EC, human iPSCs were treated with different concentrations of GSK3 inhibitor CHIR99021 for 4 days, followed by VEGF, BMP4, SB431542, as well as FGF2 for 8 days, while different ratios of differentiation medium and EMV2 were used to culture the cells. On day12, cells were purified through sorting to get hiPSC-ECs. After hiPSC-ECs were sorted they were cultured for 5 days, and they reached 90% confluency. Supplementary Figure S1 demonstrates a schematic of differentiating ECs derived from hiPSCs.

**Figure 1:**
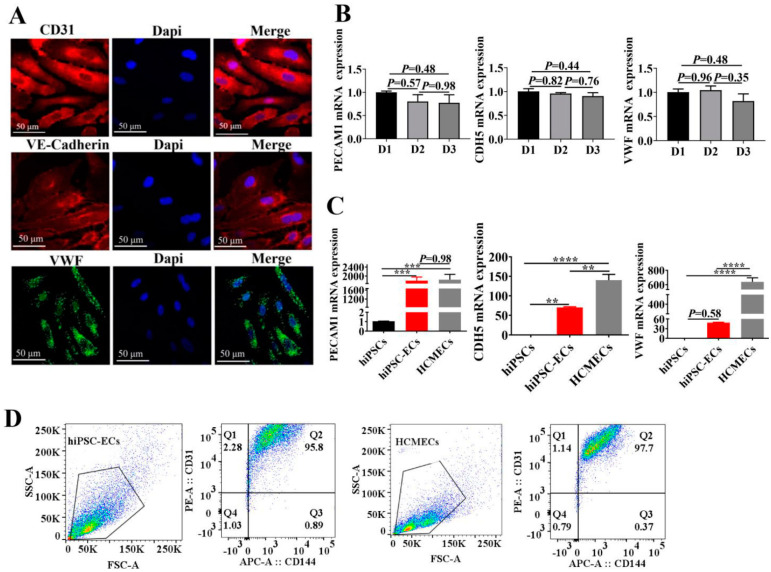
Generation and identification of endothelial cells derived from human induced pluripotent stem cells (hiPSC-ECs). (**A**) The expression of definitive endothelial markers for CD31 (PECAM1, red), VE-cadherin (CDH5, red), and von Willebrand Factor (vWF, green) in hiPSC-ECs was confirmed using immunofluorescence staining. Cell nuclei were counterstained with DAPI (blue). (**B**) The mRNA level of endothelial cells markers PECAM1, CDH5 and VWF in cells from three healthy donors (D1, D2, and D3). The results are shown as mean ± SEM (n = 3). (**C**) The expression profile of hiPSC-EC markers PECAM1, CDH5 and VWF gene expression were examined by real-time RT-PCR. The results shown are mean ± SEM (n = 3 healthy iPSC). (**D**) Flow cytometry analysis of hiPSC-EC differentiation efficiency. HiPSC-ECs and Human Cardiac Microvascular Endothelial Cells (HCMEC) were stained with the endothelial markers PECAM1 (CD31), VE-cadherin (CD144). ** *p* < 0.005, *** *p* < 0.0005, **** *p* < 0.00005.

To examine the success and efficiency of differentiation of hiPSCs into hiPSC-ECs, cells expressing CD31, VE-Caderin and VWF, markers of EC phenotype, were measured by immunofluorescence staining (Figure 1A). The results showed that the majority of cells were CD31, VE-Cadherin and VWF positive, indicating successful generation of endothelial cells from iPS cells. All the experiments described were performed in hiPSC-ECs from three healthy donors (D1, D2, D3). We performed a qPCR experiment to compare the mRNA level of endothelial markers PECAM1, CDH5, and VWF among D1, D2 and D3 (Figure 1B). However, the expression levels of some markers in hiPSC-ECs and HCMECs were different. The expression of CDH5 and VWF in hiPSC-ECs were lower compared to HCMECs (Figure 1C). In addition, to test the purity of positive iPSC-ECs, flow cytometry was performed. CD31 and VE-cadherin double positive cells were similar to that of HCMEC, suggesting a high efficiency of the differentiation protocol (Figure 1D). 

### 2.2. Gene Expression of Ion Channel and Membrane Receptors in hiPSC-ECs

**Figure 2:**
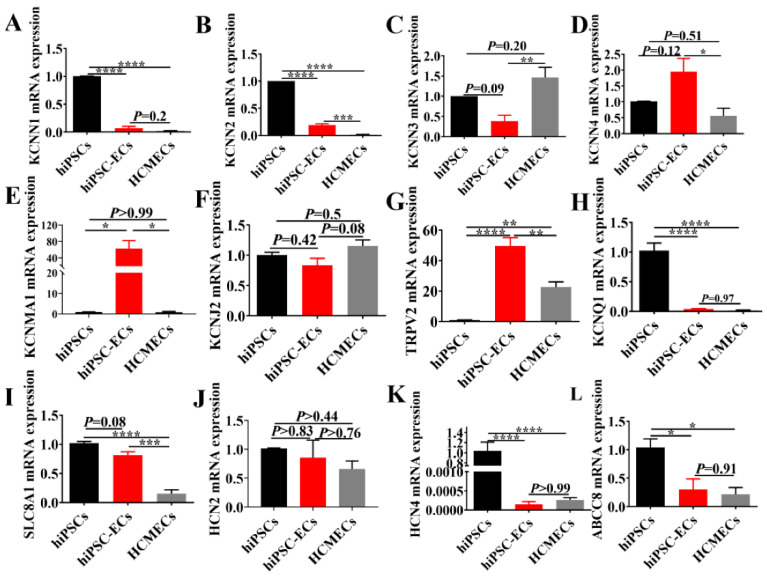
MRNA expression level of ion channels in hiPSCs, hiPSC-ECs and HCMECs. All the data are normalized to that of hiPSC values (**A**–**L**). Transcriptional levels of small conductance calcium-activated potassium channel four isoforms, including SK1 ((**A**), n = 3), SK2 ((**B**), n = 3), SK3 ((**C**), n = 3), SK4 ((**D**), n = 3), large conductance calcium-activated potassium channel (BK, (**E**), n = 3), inwardly rectifying potassium channel Kir2.1 (KCNJ2, (**F**), n = 4), transient receptor potential vanilloid 2 (TRPV2, (**G**), n = 4), slowly activated delayed rectifier potassium channel (IKs, (**H**), n = 4), Na/Ca exchanger ((**I**), n = 3), hyperpolarization-activated cyclic nucleotide-gated channels (HCN2, (**J**), n = 3, and HCN4, (**K**), n = 4) as well as ATP-sensitive potassium channel (KATP, (**L**), n = 3) were examined by qPCR. The results are presented as mean ± SEM, * *p* < 0.05, ** *p* < 0.005, *** *p* < 0.0005, **** *p* < 0.00005.

To examine the ion channel and receptor expression profile in hiPSC-ECs, the mRNA expression levels in hiPSCs, hiPSC-ECs and HCMECs were analyzed by qPCR (Figure 2). KCNN1(Potassium Calcium-Activated Channel Subfamily N Member 1, SK1), KCNN2 (Potassium Calcium-Activated Channel Subfamily N Member 2, SK2), KCNN3 (Potassium Calcium-Activated Channel Subfamily N Member 3, SK3), KCNN4 (Potassium Calcium-Activated Channel Subfamily N Member 4, SK4), KCNMA1 (Potassium Calcium-Activated Channel Subfamily M Alpha 1, BK), KCNJ2 (Potassium Inwardly Rectifying Channel Subfamily J Member 2, Kir2.1, IK1), TRPV2 (Transient Receptor Potential Cation Channel Subfamily V Member 2), KCNQ1 (Potassium Voltage-Gated Channel Subfamily Q Member 1, IKs, Kv7.1), SLC8A1 (Solute Carrier Family 8 Member A1, Na+/Ca^2+^-exchanger, NCX1), ABCC8 (ATP Binding Cassette Subfamily C Member 8, KATP, beta-subunit SUR1), HCN2 (Hyperpolarization Activated Cyclic Nucleotide Gated Potassium And Sodium Channel 2, If channel) and HCN4 (Hyperpolarization Activated Cyclic Nucleotide Gated Potassium Channel 4, If channel) were detected in hiPSCs, hiPSC-ECs and HCMECs, showing that KCNN1, KCNN2, KCNQ1, SLC8A1, HCN2, HCN4 and ABCC8 had lower expression levels in hiPSC-ECs and HCMECs than in hiPSCs (Figure 2A,B,H,I,K,L). Interestingly, comparing the expression of KCNN4 gene, KCNMA1 gene, TRPV2 gene, SLC8A1 gene and KCNN2 gene in hiPSC-ECs and HCMEC, much higher expressions were observed in the hiPSC-ECs (Figure 2B,D,E,G,I). Of note, KCNN3 gene was the most abundantly expressed in HCMECs (Figure 2C). Additionally, no statistically significant differences in the KCNN1, KCNJ2, KCNQ1, HCN2, HCN4 and ABCC8 gene expressions were observed between hiPSC-ECs and HCMECs (Figure 2A,F,H,J–L).

**Figure 3:**
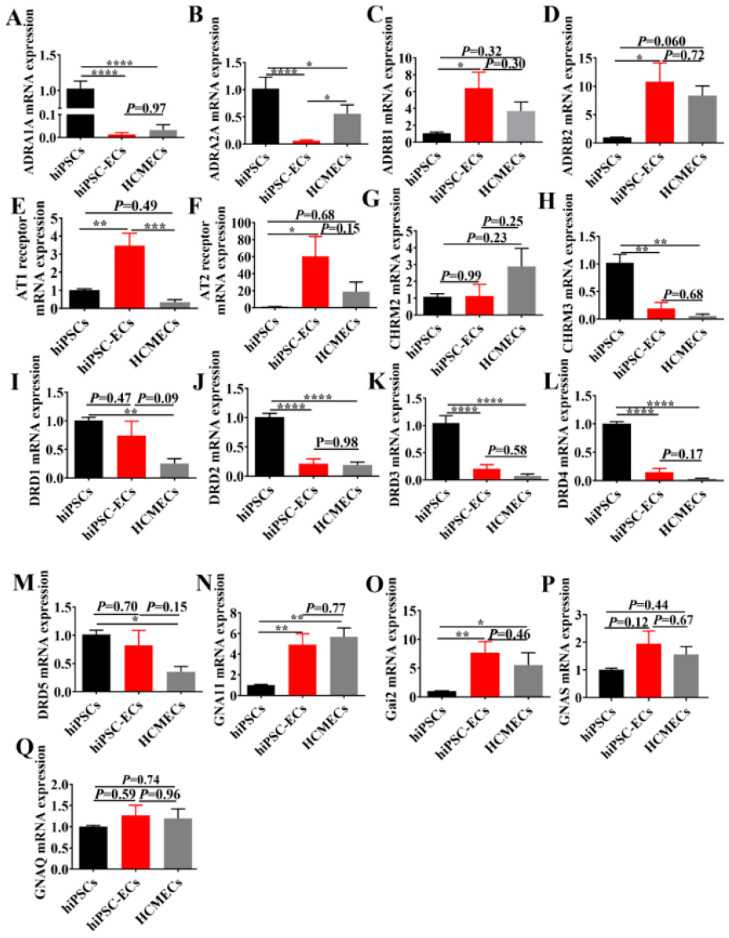
Gene expression of receptor in hiPSCs, hiPSC-ECs and HCMECs. The mRNA expression level of adrenoceptors, including ADRA1A ((**A**), n = 6), ADRA2A ((**B**), n = 6), ADRB1 ((**C**), n = 6), ADRB2 ((**D**), n = 6), angiotensin II receptor, e.g., AT1 ((**E**), n = 6) and AT2 ((**F**), n = 6), muscarinic receptors, such as CHRM2 ((**G**), n = 6) and CHRM3 ((**H**), n = 3), dopamine receptor-DRD1 ((**I**), n = 6), DRD2 ((**J**), n = 6), DRD3 ((**K**), n = 6), DRD4 ((**L**), n = 6), DRD5 ((**M**), n = 6), and G proteins, GNA11 ((**N**), n = 6), Gαi2 ((**O**), n = 6), GNAS ((**P**), n = 6) and GNAQ ((**Q**), n = 6) were analyzed by qPCR. The *p*-values were determined vs. hiPSC according to the analysis of one-way ANOVA with Bonferroni post-hoc. Results are presented as mean ± SEM, * *p* < 0.05, ** *p* < 0.005, *** *p* < 0.0005, **** *p* < 0.00005.

Additionally, we evaluated the expression of adrenergic, muscarinic, dopamine, angiotensin II receptors and G proteins. The expressions of α-adrenoceptors ADRA1A and ADRA2A in hiPSC-ECs and HCMECs were lower than those in hiPSCs (Figure 3A,B), while transcripts of β-adrenoceptors (ADRB1 and ADRB2) in hiPSC-ECs and HCMECs were highly expressed compared with hiPSCs, and there was no significant statistic difference between hiPSCs and hiPSC-ECs (Figure 3C,D). The expression level of muscarinic receptor, M3 (CHRM3), but not M2 (CHRM2), in the hiPSC-ECs and HCMECs was lower than that in hiPSC, and no significant difference between hiPSC-ECs and HCMECs was detected (Figure 3G,H). The gene expression of dopamine receptors, such as DRD1, DRD2, DRD3, DRD4 and DRD5 were detected (Figure 3I–M). The expression of DRD1–5 in hiPSC-ECs and HCMECs was lower than that of hiPSC, but no significant difference was observed between hiPSC-ECs and HCMECs. Next, the gene changes in angiotensin II receptors AT1 and AT2 were observed (Figure 3E,F), and, in particular, a higher expression level of AT1 was found in hiPSC-ECs (Figure 3F). Besides, the mRNA expression of G-proteins, e.g., Gs (GNAS), Gi (GNAI2), and Gq (GNA11 and GNAQ), were also measured, showing that the G proteins were almost equally expressed in hiPSC-ECs and HCMECs (Figure 3N–Q).

### 2.3. Membrane Potentials and Ion Channel Currents in hiPSC-ECs and HCMECs

**Figure 4:**
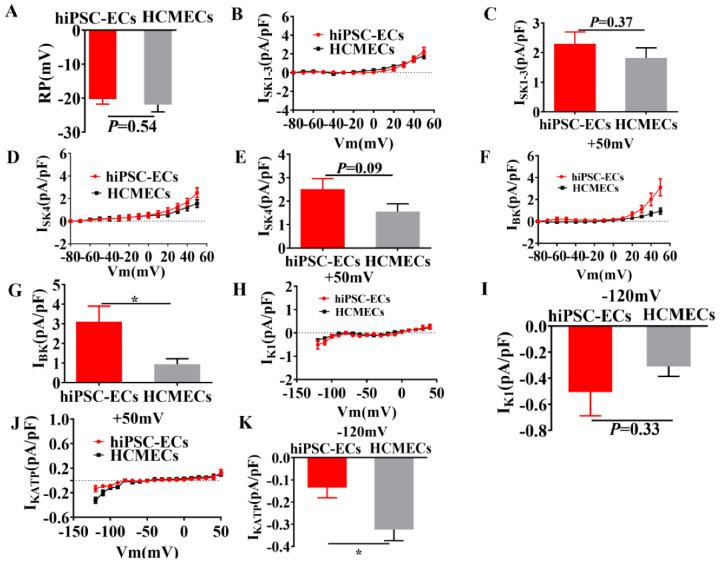
Membrane potential and ion channel currents in hiPSC-ECs and HCMECs. Membrane potential and ion channel currents were recorded by the whole cell patch clamp technique. Intracellular and extracellular solutions or specific blockers were applied to confirm the identity of expected currents. (**A**) Mean values of resting potential (RP) in hiPSC-ECs and HCMECs, n = 10 cells/group. (**B**) Apamin-sensitive SK1–3 current (I_SK1–3_) density from −80 mV to +50 mV in hiPSC-ECs and HCMECs. (**C**) The mean data of ISK1–3 at +50 mV. n = 10 cells/group. (**D**) Tram-34-sensitive current (I_SK4_) densities from −80 mV to +50 mV in hiPSC-ECs and HCMECs. (**E**) I_SK4_ density at +50 mV in hiPSC-ECs and HCMECs. n = 10 cells/group. (**F**) Iberitoxin sensitive BK currents at different potentials in hiPSC-ECs and HCMECs. (**G**) BK current density at +50 mV in hiPSC-ECs and HCMECs. n = 10 cells/group, * *p* < 0.05. (**H**) I_K1_ current density from −120 mV to +50 mV in hiPSC-ECs and HCMECs. (**I**) IK1 at −120 mV in hiPSC-ECs and HCMECs. n = 10 cells/group. (**J**) I_KATP_ current density in hiPSC-ECs and HCMECs from −120 mV to +50 mV. (K) Current density of I_KATP_ at −120 mV in hiPSC-ECs and HCMECs. n = 10 cells/group, * *p* < 0.05.

To characterize the electrophysiology property of hiPSC-ECs, we performed a whole cell patch clamp experiment to measure membrane potentials and ion channel currents, such as I_SK1–3_, I_SK4_, I_BK_, I_KATP_, I_K1_, in hiPSC-ECs and HCMECs. Figure 4A demonstrates that no significant differences in the membrane potential between hiPSC-ECs and HCMECs were detected. The small conductance calcium-activated K^+^ channel currents (I_SK1–3_) (Figure 4B,C, Supplementary Figure S2), intermediate conductance calcium-activated K^+^ channel current (I_SK4_) (Figure 4D,E and Figure S3) and inward rectifier K^+^ channel current (I_K1_) (Figure 4H,I and Figure S5) were similar in the hiPSC-ECs and HCMECs. The big conductance calcium-activated K^+^ channel current (I_BK_) in hiPSC-ECs was larger (Figure 4F,G and Figure S4), while ATP-sensitive K^+^ channel current (I_KATP_) in hiPSC-ECs was smaller than that in HCMECs (Figure 4J,K and Figure S6).

### 2.4. Functional Characterization of hiPSC-ECs

**Figure 5:**
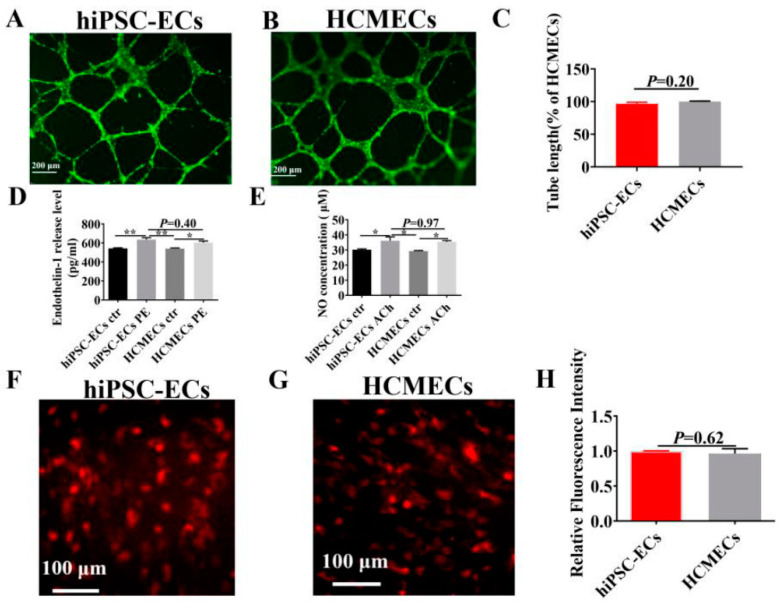
Comparison of the functional characteristics of endothelial cells in hiPSC-ECs and HCMECs. The triplicate tube formation experiment was carried out. Representative images of tube formation are shown (**A**,**B**). The scale bar was 200 μm. (**C**) Tube length of formed tubes in hiPSC-ECs and HCMECs. (**D**) ET-1 levels in response to phenylephrine (PE) were measured using the Elisa method in hiPSC-ECs and HCMECs. n = 4, * *p* < 0.05; ** *p* < 0.005. (**E**) NO release was measured in hiPSC-ECs and HCMECs treated with acetylcholine (Ach). n = 4, * *p* < 0.05. (**F**,**G**) Fluorescence staining showing the degree of LDL uptake in hiPSC-ECs and HCMECs. Scale bar was 100 μm. (**H**) Relative fluorescence intensity of LDL in hiPSC-ECs and HCMECs. All data shown are mean ± SEM. n = 3, * *p* < 0.05; ** *p* < 0.005.

To assess the functions of hiPSC-ECs, we investigated the tube formation of hiPSC-ECs and HCMECs, which reflect properties relevant to angiogenesis. Figure 5A–C demonstrates that there was no statistically significant difference in tube formation ability between hiPSC-ECs and HCMECs. Next, we employed phenylephrine (PE) and the endothelium-dependent dilator, acetylcholine (ACh) to assess the endothelial cell-related vasoconstricting and vasodilating functions. Figure 5D,E show that PE enhanced the release of the endogenous vasoconstrictor endothelin-1 (ET-1) in hiPSC-ECs and HCMECs by a similar amount. ACh similarly increased nitric oxide content in hiPSC-ECs and HCMECs. Furthermore, the degree of LDL uptake in hiPSC-ECs and HCMECs was not significantly different (Figure 5F–H). The results revealed that hiPSC-ECs and HCMECs were similar in terms of tube formation, vasoconstriction and relaxation response, as well as in LDL uptake, i.e., they had similar endothelial functions.

**Figure 6:**
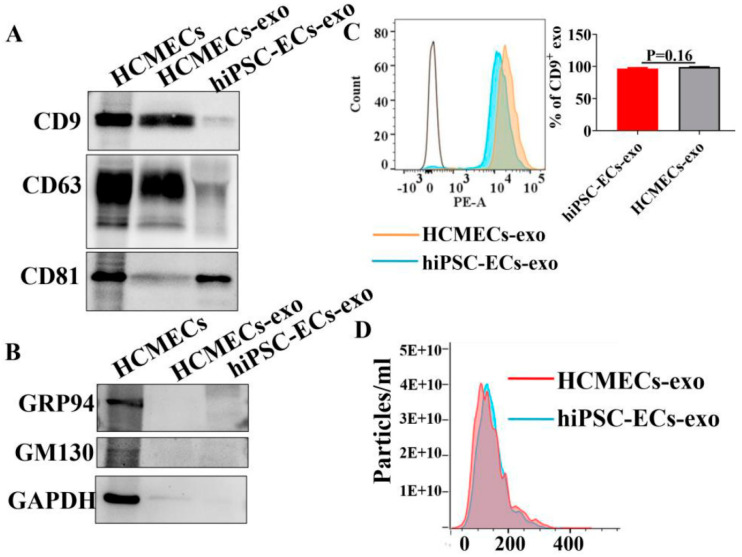
Characterization of the purified exosomes from hiPSC-EC and HCMEC conditioned media. Exosomes isolated from cell culture media of hiPSC-ECs and HCMECs. (**A**) Western blot showed the detection of exosome markers CD9, CD63, and CD81 in isolated exosomes. (**B**) Glucose-regulated protein 94 (GRP94) and Golgi marker GM130 were not found in exosomes. (**C**) Flow cytometry analysis using exosome marker CD9. (**D**) The size distribution of isolated exosomes was measured by nanoparticle tracking analysis (NTA) in hiPSC-ECs and HCMECs.

Exosomes are one of the most critical paracrine factors for stem cells to control the reprogramming of injured cells [21]. Therefore, we investigated the ability of hiPSC-ECs and HCMECs to secrete exosomes. First, exosomes were extracted from both hiPSC-ECs and HCMECs, then western blot analysis for exosome markers, CD9, CD63, and CD81, was performed (Figure 6A). The expression levels of CD9 and CD63 were higher in HCMECs, whereas the expression of CD81 was higher in hiPSC-ECs (Figure 6A). To examine if cellular proteins were present in exosomes, we also tested glucose-regulated protein 94 (GRP94) and Golgi marker GM130 by western blot. Figure 6B demonstrates that both proteins were present in the endothelial cells but were absent in the exosomal samples, indicating that high purity exosomes were extracted from hiPSC-ECs and HCMECs. Next, flow cytometry was carried out to detect CD9 expression on the exosomes from hiPSC-ECs and HCMECs. We observed that 99% of exosomes derived from HCMECs and 95% of exosomes extracted from hiPSC-ECs were positively stained, respectively (Figure 6C). Finally, the numbers of exosomes extracted from hiPSC-ECs and HCMECs were quantitatively analyzed, showing no significant difference between both cell types (Figure 6D).

## 3. Discussion

In this study, we, for the first time, investigated the electrophysiological and functional properties of hiPSC-ECs and compared them to those of HCMECs.

HiPSC-ECs may be useful for disease modeling, cell replacement therapy and drug screening [4]. Our results demonstrated that hiPSC-ECs have been successfully differentiated from hiPSCs by showing expression of EC markers PECAM1, CDH5 and VWF and endothelial properties. Notably, the efficiency of this protocol for obtaining CD31 and VE-Cadherin double positive cells was 95.8%, which was almost the same as HCMECs. To date, several methods of differentiating ECs derived from hiPSC have emerged [7,31,32]. Each method has differences and similarities. One advantage of using hiPSC-ECs in studies is to provide unlimited cell sources. Another advantage is that patient-specific hiPSC-ECs can be generated to model disease-specific abnormalities.

Ion channels which are regulated by shear force have an influence on endothelial function [24,33]. Ion channels play a crucial role in regulating intercellular permeability, EC proliferation, and angiogenesis [34]. So far, the expression and characterization of ion channels in hiPSC-ECs have not been reported. To clarify the similarities and differences between hiPSC-ECs and native ECs, to determine the usefulness and importance of hiPSC-ECs in endothelial researches, we employed qPCR to study ion channels in our hiPSC-ECs and compared them with HCMECs. The expression of SK1 channels in isolated and cultured ECs were rarely detected, while the mRNA of SK2 channels were easily detectable [35,36,37,38]. Our study demonstrated that the expression of KCNN4 (SK4) gene, KCNMA1 (BK) gene, TRPV2 gene, SLC8A1 gene and KCNN2 (SK2) gene in hiPSC-ECs exhibited a much higher expression than HCMECs. Of note, the KCNMA1 gene was the most abundantly expressed in hiPSC-ECs. These data provided evidence that hiPSC-ECs may possess ion channel expression profiles different from native endothelial cells. K^+^ channels in ECs regulate vascular tones by influencing release of NO and endothelium-derived hyperpolarizing factor [39]. Membrane potential (RP) is determined by K^+^ channel currents and is an essential regulator for regulating intracellular and extracellular signal transduction [40]. RP in ECs modulates NO production and eNOS activity [40]. Taken together, ion channel currents may contribute to NO generation via changing RP. In this study, no significant difference in RP between hiPSC-ECs and HCMECs was observed. Currently, only several channel currents in ECs have been investigated, mainly focusing on I_SK1–3_, I_SK4_, I_BK_, I_K1,_ and, I_KATP_ [27,35,38,41,42,43,44,45,46,47], which were not studied in hiPSC-ECs. Thus, in our study, we mainly compared the current density of I_SK1–3_, I_SK4_, I_BK_, I_K1,_ and I_KATP_ in hiPSC-ECs with that of HCMECs. We found that I_BK_ in hiPSC-ECs was higher than that of HCMECs, while I_KATP_ in hiPSC-ECs was smaller than that in HCMECs and no significant differences were observed between hiPSC-ECs and HCMECs in I_SK1–3_, I_SK4_ and I_K1_.

Importantly, the RP in hiPSC-ECs and HCMECs was very similar, suggesting that the basal electrical property in hiPSC-ECs did not differ from that in native endothelial cells, although some ion channel expression and currents differed quantitatively. The reason for the unchanged RP could be the reduction of I_KATP_ and increase of I_BK_ because the effects of both changes on membrane potential can be counteractive. Another possibility could be that the changed ion channel currents had a minor influence on the membrane potential.

All reported hiPSC-ECs showed common features of endothelial cells, such as endothelial surface marker expression, tube formation and the capacity for LDL uptake [48]. We investigated the tube formation and LDL uptake of our hiPSC-ECs to assess the endothelial functions in hiPSC-ECs. By comparing with HCMECs, no significant difference between them could be detected, which is indicative of hiPSC-EC functions being similar to that of native endothelial cells. NO is the major vasodilator produced by ECs, which is closely related to the ability of endothelium to respond to mechanical shear stress [49,50]. Arterial endothelial cells (AECs) were found to have a greater NO production rate than HUVECs but were similar to primary human coronary arterial endothelial cells (HCAECs) [5]. ET-1, a powerful vasoconstrictor, is a vasoactive peptide released by endothelial cells [51,52]. The generation of NO and ET-1 in endothelium is important for the vessel tone. Our study demonstrated that NO production stimulated by Ach and ET-1 release caused by PE stimulation were similar between hiPSC-ECs and HCMECs. This again indicates that the hiPSC-ECs possess functional properties similar to native endothelial cells. We further demonstrated, in the present study, that hiPSC-ECs secreted exosomes as HCMECs did. It has been reported that exosomes derived from hiPSC-ECs play a profitable role in treating ischemic injury, which has become an attractive alternative to cell therapy [21,53]. It is possible to develop and implement new methods that rely on exosomes as therapeutic targets to treat various diseases [54]. Our study confirmed that high purity exosomes can be extracted from hiPSC-ECs and can provide an alternative for exosome-related studies. Nevertheless, exosomes from hiPSC-ECs and HCMECs presented different sensitivity to markers CD9, CD63, and CD81, which may also provide relevant information for future studies with respect to exosomes.

Adrenaline and acetylcholine act on endothelial cells to regulate vascular tone [55]. G protein-coupled receptor (GPCR) signaling pathways contribute to endothelial dysfunction [56]. AT1 receptor signaling mechanism was involved in oxygen radical production and caused P-selectin- and PSGL-1-mediated platelet-leukocyte-endothelial cell interactions [57]. Ang-II induced apoptosis of human right and left ventricular endocardial endothelial cells via AT_2_ receptors [58]. Dopamine acted on D2 receptors and inhibited angiotensin II-mediated angiogenesis by inhibiting the expression of AT1R in ECs [59]. Ach, which is a prototype muscarinic receptor agonist, was related to endothelium-dependent relaxation of arteries in vitro [55,60]. The muscarinic acetylcholine receptor (AChMR1–5) is the basis of the cellular response after ACh is released [60]. The expression profile of these receptors has not been examined in hiPSC-ECs. Our data showed that the mRNA expression of ADRA1A, ADRB1, ADRB2, AT2, CHRM2, CHRM3, DRD1, DRD2, DRD3, DRD4, DRD5, and the receptor coupled G-proteins GNA11, Gai2, GNAS, and GNAQ in the hiPSC-ECs were not significantly different from those of the HCMECs, indicating that hiPSC-ECs are suitable for studies relating to these receptors and their signaling. The expression of AT1 and AT2 receptor in hiPSC-ECs was higher than that of HCMECs, which should be considered when studies relating to these receptors are carried out. Of note, we observed similar responses of hiPSC-ECs and HCMECs to PE and Ach, showing similar production of NO and ET-1. This implies that the functional signaling in both types of cells is similar and hiPSC-ECs can be a substitute for native endothelial cells, at least regarding adrenoceptor/muscarinic receptor and NO/ET signaling.

We only selected one of several differentiation schemes to obtain hiPSC-ECs and compared them with HCMECs in terms of electrophysiological properties, receptor expression, tube formation, LDL uptake and the ability to secrete exosomes. Therefore, the possibility that different differentiation protocols may generate endothelial cells with different properties cannot be excluded. Furthermore, we did not measure all the ion channel currents and all possible receptors and their signaling. Probably, there are many other similarities or differences between hiPSC-ECs and native endothelial cells, which may be necessary for endothelial studies and need to be clarified.

## 4. Materials and Methods

### 4.1. Ethics Statement

The skin biopsies from three healthy donors were obtained with written informed consent. The study was approved by the Ethics Committee of the Medical Faculty Mannheim, Heidelberg University (approval number: 2018-565N-MA) and by the Ethics Committee of the University Medical Center Göttingen (approval number: 10/9/15). The study was carried out in accordance with the approved guidelines and conducted in accordance with the Helsinki Declaration of 1975 (https://www.wma.net/what-we-do/medical-ethics/declaration-of-helsinki/, accessed on 30 May 2022), revised in 2013.

### 4.2. Generation of Human-Induced Pluripotent Stem Cells

Human iPSC lines from three healthy donors were used in this study. The first hiPSC line UMGi014-B clone 1 (ipWT1.1, here abbreviated as D1) was generated from dermal fibroblasts of a male donor, reprogrammed using integration-free episomal 4-in-1 CoMiP reprogramming plasmid (Addgene, Watertown, MA, USA, #63726) with the reprogramming factors OCT4, SOX2, KLF4, c-MYC and short hairpin RNA against p53, and described previously [61]. The second hiPSC line UMGi130-A clone 5 (isWT11.5, here abbreviated as D2) was generated from peripheral mononuclear blood cells of a male donor, reprogrammed using integration-free Sendai virus with the reprogramming factors OCT4, SOX2, KLF4, and c-MYC and applied previously [62]. The third hiPSC line UMGi124-A clone 11 (isVHFx-R1.11, here abbreviated as D3) was generated from dermal fibroblasts of a male donor, reprogrammed using integration-free Sendai virus with the reprogramming factors OCT4, SOX2, KLF4, and c-MYC and applied previously [62].

### 4.3. Cell Culture

The hiPSCs (over passage 20) from the three healthy individuals were seeded onto culture dishes and well coated with Matrigel (Corning, 354230) at a 1:100 dilution and grown for 3–4 days until they reached ~75% confluence. The culture medium TeSR-E8 (Stem cell Technologies, 05990), supplemented with Essential 8 Supplement, was used for hiPSCs. Human Cardiac Microvascular Endothelial Cells (HCMEC) (Promocell, Heidelberg, Germany, C-12285) from 3 different donors were cultured in Endothelial Cell Growth Medium MV2 (EMV2) supplemented with SupplementMix (Promocell, Heidelberg, Germany, C-22022).

### 4.4. Generation of hiPSC-ECs

The method for differentiating hiPSC-ECs was modified from previously described methods [6]. The differentiation medium (RPMI (Gibco, Waltham, MA, USA, 61870044) and B-27 Supplement without Insulin (ThermoFisher Scientific, Waltham, MA, USA, A1895601), supplemented with 6 μM glycogen synthase kinase 3-β inhibitor CHIR-99021 (StemMACS, 130-103-926), was used for hiPSCs to initiate endothelial cell differentiation on day 0 and 2 μM CHIR-99021 on day 2. From day 4 to day 12, different combinations of differentiation media and EMV2 supplemented with SupplementMix (100% differentiation medium on day 4, 50% differentiation medium and 50% EMV2 on day 6, 25% differentiation medium and 75% EMV2 medium on day 8, and 100% EMV2 medium on day 10) with growth factors, that included 50 ng/mL VEGF (Promocell, Heidelberg, Germany, C-64420), 20 ng/mL FGF2 (Miltenyi Biotec, Bergisch Gladbach, Germany, 130-093-841), and 20 ng/mL BMP4 (R&D Systems, Minneapolis, MN, USA, 314-BP-010), 10 μM SB431542 (Selleckchem, Houston, TX, USA, S1067), were used to culture cells. At day 12 post-differentiation, the expression of different EC markers was assessed to prove the successful differentiation of hiPSC-ECs and flow cytometry was used to purify the hiPSC-ECs. HiPSC-ECs from passage 2 to passage 4 were used for PCR, western blot, ELISA, FACS and patch clamp studies. Data from hiPSC-ECs from the three hiPSC cell lines were combined for analyses.

### 4.5. Identification of HCMECS and hiPSC-ECs

At day 12 post-differentiation, all differentiated ECs were harvested after trypsin-EDTA digestion and washed in cold PBS three times. Pellets were resuspended in 0.4% *v*/*v* bovine serum albumin (BSA) and were standardized to a concentration of 1 × 10^6^ cells/mL. Cell suspensions were directly-labeled with PE anti-human CD31 antibody (Biolegend, San Diego, CA, USA, 303106) and Alexa Fluor^®^ 647 anti-human CD144 (VE-Cadherin) antibody (Biolegend, San Diego, CA, USA, 8514). After 1 h incubation at 37 °C, hiPSC-ECs and HCMEC were centrifuged and supernatants decanted and then resuspended in ice-cold FACS buffer for live cell sorting every time. For the identification of hiPSC-ECs, cells were incubated for 1 h at 37 °C and measured by BD FACSCanto™ II (Becton Dickinson, Heidelberg, Germany). Data analysis were conducted using the BD FACS Diva software (Version 8.0.1).

### 4.6. Immunofluorescence (IF) Staining

HiPSC-ECs and HCMEC were washed with PBS 3 times and then fixed with 4% Paraformaldehyde (PFA, Sigma, Burlington, MA, USA) at RT for 15 min, permeabilized with 0.1% Triton-X100 (Carl Roth) for 20 min, blocked with 5% bovine serum albumin (BSA, Sigma-Aldrich) in PBS for 1 h. After that, cells were incubated with primary antibodies anti-CD31 (Abcam, Cambridge, UK, ab32457), anti-VE-cadherin (Cell Signaling Technology, 2500) and anti-Von Willebrand Factor (vWF, Abcam, ab194405) overnight at 4 °C. Cells were then washed with PBS and incubated for 1 h at room temperature with corresponding AlexaFluor conjugated 488 and 647 secondary antibodies (ThermoFisher). Cells were then washed with PBS, and incubated with DAPI (Biozol, Eching, Germany, H-1200) for 10 min at RT in the dark. Images were collected using the Confocal Microscope TCS SP-8 upright (Leica, Germany) with Plan-Apochromat 40×/0.6 objective.

### 4.7. Polymerase-Chain-Reaction Assays

Total RNA was extracted from hiPSC, hiPSC-ECs and HCMEC, using the RNeasy Mini kit (Qiagen, Hilden, Germany, 74106) and 500 ng of RNA was reverse transcribed using a high-capacity cDNA reverse transcription kit (Thermo Fisher, 4368814), according to manufacturers’ recommendations. The procedure was performed with the following cycle conditions: 10 min at 25 °C, 120 min at 37 °C, 5 min at 85 °C and store at 4 °C. Quantitative reverse-transcription polymerase chain reaction (qRT-PCR) was performed on the StepOne Plus Real-Time PCR platform (Applied Biosystems, Waltham, MA, USA) using HotRox Master Mix (BIORON life science, Römerberg, Germany). Cycle Conditions were as follows: 94 °C for 2 min; 94 °C for 10 s, 60 °C for 20 s, 72 °C for 1 min, 40 cycles, followed by a melting curve analysis. Relative mRNA expression level was calculated as the expression of the mRNA of the gene of interest relative to GAPDH in samples from treated or untreated (control) cells, which was calculated by the ΔΔCT method, based on the threshold cycle (CT), as fold change = 2-Δ(ΔCT), where ΔCT = CTtarget gene − CTGAPDH and Δ(ΔCT) = ΔCTtreated − ΔCTcontrol. The list of gene specific primers used in this study is available in Supplemental Tables S1 and S2.

### 4.8. Enzyme-Linked Immunosorbent Assay (ELISA)

The enzymatic activity and content of endothelin-1 (ET-1) was tested by enzyme-linked immunosorbent assay (RayBiotech, Peachtree Corners, GA, USA, ELH-EDN1-1) and nitric oxide (NO) release level was measured by Nitric Oxide Assay Kit (Thermo Fisher, EMSNO). The supernatant of the culture media from HCMECS and hiPSC-ECs were collected to determine the levels of NO and ET-1, according to the manufacturer’s instructions.

### 4.9. Vascular Tube Formation Assay

After coating the 24-well plate with Corning^®^ Matrigel^®^ Basement Membrane Matrix (Corning), sorted hiPSC-ECs and HCMECs were seeded at 5 × 104 cells per well (24-well plate) in EGMV2 medium. After 16 h incubation, capillary network images were captured using a Leica microscope and quantitation analysis was made using ImageJ.

### 4.10. Low Density Lipoprotein (LDL) Uptake Assay

HiPSC-ECs and HCMECs at densities of 1 × 105/well were seeded in 24-well cell culture plates. After washing three times with PBS, hiPSC-ECs and HCMECs were incubated with LDL-DyLight™ 550 (Abcam, ab133127) in the EMV2 medium at 37 °C for 4 h. Then, they were washed with PBS for three times, replaced with PBS and observed with a fluorescence microscope with filters capable of measuring excitation and emission wavelengths 540 and 570 nm, respectively.

### 4.11. Patch-Clamp

Standard patch-clamp recording techniques were used to measure the small conductance calcium-activated potassium current (I_SK1–3_), intermediate conductance calcium-activated potassium channel current (I_SK4_), large conductance calcium activated potassium current (I_BK_), adenosine triphosphate (ATP)-sensitive potassium (I_KATP_) and inward rectifier K^+^ channel current (I_K1_) in the whole-cell configuration at room temperature. Different protocols were employed for measuring different currents. To isolate one type of ion channel current from others, a specific channel blocker or solution was used. The resting membrane potential (RP) was recorded with whole cell current-clamp mode.

The bath solution contained 130 mmol/L NaCl, 5.9 mmol/L KCl, 2.4 mmol/L CaCl_2_, 1.2 mmol/L MgCl_2_, 11 mmol/L glucose, and 10 mmol/L HEPES (pH = 7.4 (NaOH)). The pipette solution contained 10 mM HEPES, 126 mM KCl, 6 mM NaCl,1.2 mM MgCl_2_, 5 mM EGTA, 11 mM glucose, and 1 mM MgATP (pH = 7.4 (KOH)). For measuring I_SK1–3_, I_SK4_ and I_BK_, appropriate CaCl_2_ was added to the pipette solution to get the free Ca^2+^ concentration of 0.5 μM according to the calculation by the software MAXCHELATOR (http://web.stanford.edu/~cpatton/downloads.htm, accessed on 30 May 2022). For measurements of I_KATP_, the ATP-free pipette solution was used.100 nM apamin, 1 μM Tram-34 and 100 nM iberitoxin, 10 μM glibenclamide and 100 mM BaCl_2_ were added in the bath perfusion solution to block I_SK1–3_, I_SK4_ and I_BK_, I_KATP_ and I_K1_, respectively. Blocker-sensitive currents were analyzed.

### 4.12. Isolation and Quantification of Exosomes

The cells were cultured for 48 h with EMV2 medium containing 10% exosome-free serum and reached about 90% confluence. The isolation of exosomes was performed by ultracentrifugation as previously described [63]. Briefly, the supernatant from cells was collected and processed for gradient centrifugation at 200× *g* for 10 min, 2000× *g* for 10 min and 10,000× *g* for 30 min, respectively. Subsequently, the resultant supernatants were used for ultracentrifugation at 100,000× *g* for 70 min to pellet exosomes. After washing with PBS, the pellets were ultracentrifuged at 100,000× *g* for 70 min to harvest exosomes. The size and concentration of exosomes were measured by ZetaView instrument (Particle Metrix, Meerbusch, Germany) at a dilution of 1000, sensitivity 80, shutter 100. The data were analyzed with the FlowJo Software (FlowJo LLC, Ashland, OR, USA).

### 4.13. Flow Cytometry Analysis for Exosomes Derived from HCMECS and hiPSC-ECs

CD63+ exosomes were isolated from the collected EMV2 medium and then stained for flow cytometry, according to ExoStep Cell Culture (Immunostep, ExoS-25-C9) recommendations. The primary detection antibody was added to the bead-bound exosomes tube and incubated in the dark 60 min at 2–8 °C. After that, the magnetic beads were collected and then the supernatant was removed. The results were measured by BD FACS Canto™ II (Becton Dickinson, Heidelberg, Germany). Data analysis was conducted using the BD FACS Diva software (Version 8.0.1).

### 4.14. Western Blot Analysis

Total protein was extracted from exosomes with procedures as described in detail elsewhere [64]. Briefly, equal amounts of proteins were added on 4–12% SDS gels for electrophoresis and then transferred to PVDF membranes (ThermoFisher Scientific). The membranes were blocked for 1 h in 5% milk. All the membranes were incubated with primary antibodies at 4 °C overnight. Primary antibodies used in this study were as follows: rabbit-anti-CD9 (Thermo Fisher Scientific, PA5-11559), mouse-anti-CD63 (Thermo Fisher Scientific, 10628D), mouse-anti-CD81 (Thermo Fisher Scientific, MA5-13548), rabbit-anti-GRP94 (Abcam, 13509), mouse-anti-GM130 (Santa Cruz, sc-55591), mouse-anti-GAPDH (HyTest, 5G4MAb6C5). On the next day, the membranes were washed three times for 10 min in TBST (pH = 7.4) and incubated for 1 h with secondary antibodies at room temperature. Secondary antibodies used were anti-Mouse IgG (Fab specific)-Peroxidase antibody produced in goat (Sigma-Aldrich, A3682) and anti-Rabbit IgG (whole molecule)-Peroxidase antibody produced in goat (Sigma-Aldrich, A0545). The results were visualized with chemiluminescence, and the density of the bands was analyzed by ImageJ software.

### 4.15. Statistical Analysis

All the experimental data were analyzed using InStat© (GraphPad, San Diego, CA, USA) and shown as mean ± standard error (SEM). Student’s *t* test and one-way analysis of variance with Bonferroni post-hoc test were used to compare the statistical differences between different groups and among three or more groups, respectively. The *p* values < 0.05 were considered statistically significant.

## 5. Conclusions

HiPSC-ECs possess similarities, with some differences, when compared with HCMECs in the aspects of ion channel expression, receptor expression, tube formation, LDL uptake, ion channel currents and the ability to secrete exosomes. HiPSC-ECs may provide an attractive model to study the pathophysiology of endothelial dysfunction in CVD or to test drug effects.

## Data Availability

The data presented in this study are available on request from the corresponding author.

## Supplementary Materials

The supporting information can be downloaded at: https://www.mdpi.com/article/10.3390/ijms23158507/s1.
