# Supplementary material for: Functional Characterization of Human Induced Pluripotent Stem Cell-Derived Endothelial Cells
Source: Int J Mol Sci. 2022 Jul 31;23(15):8507. doi: 10.3390/ijms23158507 (PMC9368986; doi:10.3390/ijms23158507)
Supplement: Supplementary file 1 [file ijms-23-08507-s001.zip › ijms-1775294-supplementary.pdf]

**Supplementary material**

**Functional Characterization of Human Induced Pluripotent Stem Cell-Derived Endothelial Cells**

Xuehui Fan[1,2,3], Lukas Cyganek[4,5], Katja Nitschke[6], Stefanie Uhlig[7], Philipp Nuhn[6]，Karen Bieback[7], Daniel Dürschmied[1,3], Ibrahim El-Battrawy[1,3,9], Xiaobo Zhou[1,2,3*] and Ibrahim Akin[1,3]

[1]Department of Cardiology, Angiology, Hemostaseology and Medical Intensive Care, Medical Faculty Mannheim, University Medical Centre Mannheim (UMM), Heidelberg University, 68167 Mannheim, Germany; Xuehui.Fan@medma.uni-heidelberg.de (X.F.); daniel.duerschmied@medma.uni-heidelberg.de(D.D.); Ibrahim.elbattrawy2006@gmail.com (I.E.-B.); Ibrahim.Akin@umm.de (I.A.)

[2]Key Laboratory of Medical Electrophysiology, Ministry of Education and Medical Electrophysiological Key Laboratory of Sichuan Province, Collaborative Innovation Center for Prevention of Cardiovascular Diseases, Institute of Cardiovascular Research, Southwest Medical University, 646000 Luzhou, Sichuan, China

[3]European Center for AngioScience (ECAS) and German Center for Cardiovascular Research (DZHK) partner site Heidelberg/Mannheim, 68167 Mannheim, Germany

[4]DZHK (German Center for Cardiovascular Research), Partner Site, 37075 Göttingen, Germany

[5]Stem Cell Unit, Clinic for Cardiology and Pneumology, University Medical Center Göttingen，37075 Göt-tingen, Germany；lukas.cyganek@gwdg.de

[6]Department of Urology and Urosurgery, Medical Faculty Mannheim, Heidelberg University, 68167 Mannheim, Germany；katja.nitschke@umm.de (K.N.)；philipp.nuhn@ medma.uni-heidelberg.de（P.N.）

[7]Flow Core Mannheim Medical Faculty Mannheim, Heidelberg University , 68167 Mannheim, Germany; Stefanie.Uhlig@medma.uni-heidelberg.de (S.U.) ; Karen.Bieback@medma.uni-heidelberg.de (K.B.)

[8]Instituete of Transfusion Medicine and Immunology, Medical Faculty Mannheim, Heidelberg University, 68167 Mannheim, Germany

[9] Bergmannsheil Bochum, Medical Clinic II, Department of Cardiology and Angiology, Ruhr University, 44789 Bochum, Germany

**Address for correspondence**:
Xiaobo Zhou, MD.
First Department of Medicine, University Medical Centre Mannheim,
Theodor-Kutzer-Ufer 1-3, 68167 Mannheim, Germany.
Phone: 0049-621-383-1448. Fax: 0049-621-383-1474.
E-mail: xiaobo.zhou@medma.uni-heidelberg.de.

**Supplementary Table S1. List of genes, RefSeq numbers and primers for qPCR.**

| Gene symbol | RefSeq No. | Company |
| --- | --- | --- |
| ABCC8 (KATP, beta-subunit SUR1) | NM_000352 | Qiagen |
| ADRA1A | NM_033303 | Qiagen |
| ADRA2A | NM_000681 | Qiagen |
| ADRB1 | NM_000684 | Qiagen |
| ADRB2 | NM_000024 | Qiagen |
| CHRM2 | NM_000739 | Qiagen |
| CHRM3 | NM_000740 | Qiagen |
| HCN2 | NM_001194 | Qiagen |
| HCN4 | NM_005477 | Qiagen |
| KCNN2 (SK2) | NM_021614 | Qiagen |
| KCNN4 | NM_002250 | Qiagen |
| KCNQ1 ($I_{Ks}$, Kv7.1) | NM_000218 | Qiagen |
| SLC8A1 (NCX1) | NM_021097 | Qiagen |
| TRPV2 | NM_016113 | Qiagen |
| KCNJ2 | NM_000891 | Qiagen |
| KCNMA1 | NM_002247 | Qiagen |
| ADRA1A | NM_033303 | Qiagen |
| ADRA2A | NM_000681 | Qiagen |
| ADRB1 | NM_000684 | Qiagen |

| | | |
|---|---|---|
| ADRB2 | NM_000024 | Qiagen |
| CHRM2 | NM_000739 | Qiagen |
| CHRM3 | NM_000740 | Qiagen |
| DRD1 | NM_000794 | Qiagen |
| DRD2 | NM_000795 | Qiagen |
| DRD3 | NM_033660 | Qiagen |
| DRD4 | NM_000797 | Qiagen |
| DRD5 | NM_000798 | Qiagen |
| GNAS | HP101598 | Sino Biological |

**Supplementary Table S2. Primer sequences used**

| Gene | Forward primer (5′-3′) | Reverse primer (5′-3′) |
|---|---|---|
| KCNN1 | CAGCATCTCCTCCTGGATCAT | GCTGGTCACTTCCTGCTTGTC |
| KCNN3 | GCCAACACTCTGGTGGACCT | GTTGAAGCTGGCGGTGAGAT |
| AT1 receptor | GCCCTTTGGCAATTACCTATGT | CGTGAGTAGAAACACACTAGCGT |
| AT2 receptor | CCGCATTTAACTGCTCACACA | ATCATGTAGTAGAGAACAGGAATT G CTT |
| GNA11 | GATCCTCTACAAGTACGAGCAG AAC | ACTGATGCTCGAAGGTGGTC |
| Gai2 | CTTGTCTGAGATGCTGGTAATG G | CTCCCTGTAAACATTTGGACTTG |
| GNAQ | GACTACTTCCCAGAATATGATG GAC | GGTTCAGGTCCACGAACATC |
| PECAM1 | ATTGCAGTGGTTATCATCGGAG TG | CTCGTTGTTGGAGTTCAGAAGTGG |
| CDH5 | AGACACCCCCAACATGCTAC | GCAAACTCTCCTTGGAGCAC |
| VWF | GGGGTCATCTCTGGATTCAAG | TCTGTCCTCCTCTTAGCTGAA |

**Figure legends**

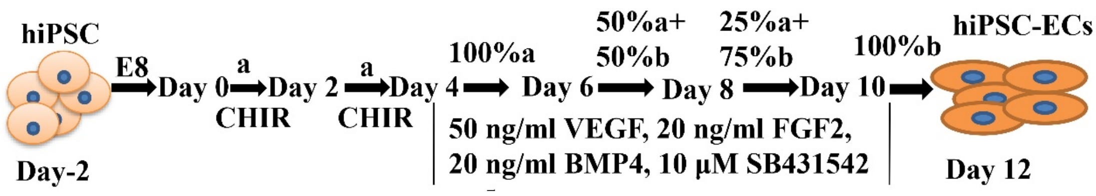


**Supplementary Figure S1: Schematic of hiPSC-ECs differentiation.** hHiPSCs were seeded and maintained in E8 medium to establish single colonies (day -2 to day 0). hHiPSCs were exposed for four days to E8 medium supplemented with different concentration of CHIR (day 0 to day 4). hHiPSCs were then exposed for eight days to different ratio of differentiation medium and EMV2 supplemented with different combinations of cytokines as indicated (day 4 to day 12) and subsequently analyzed.

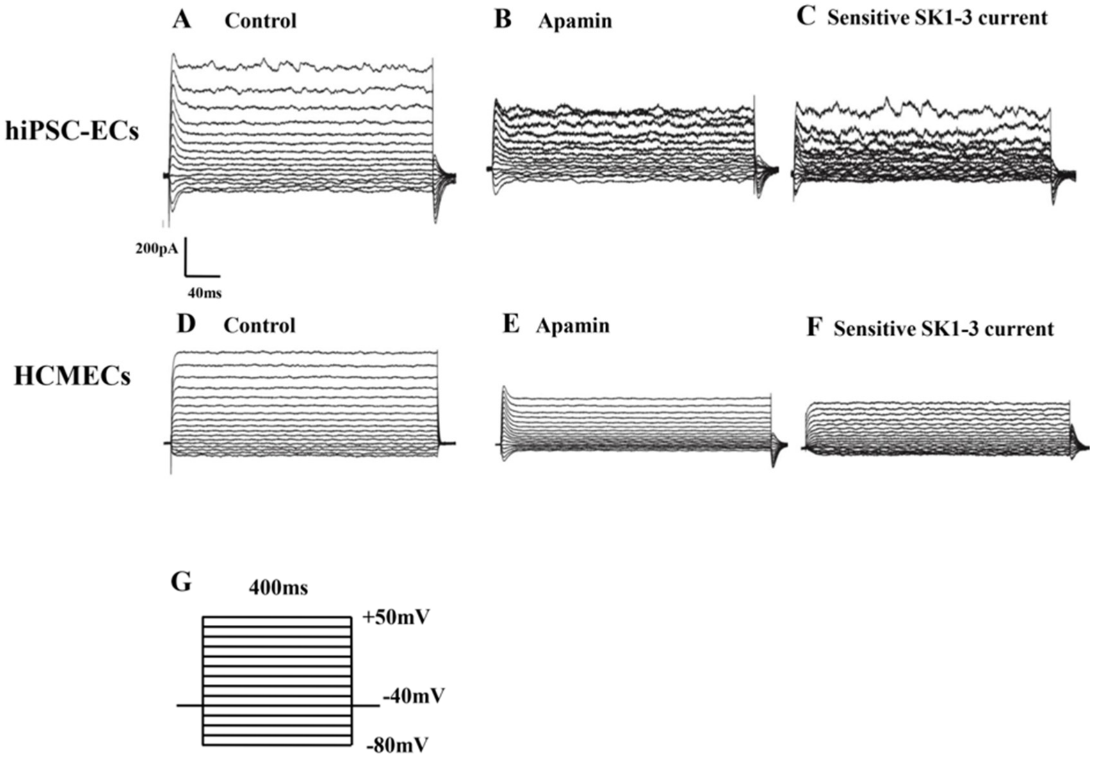


**Supplementary Figure S2. SK1-3 currents in hiPSC-ECs and HCMECs.** Membrane currents were recorded using the protocol shown in G. Apamin (100 nM), a specific blocker of SK1-3, was used to separate $I_{SK1\text{-}3}$ from other currents. (A) Representative current traces in the absence of apamin (control) in hiPSC-ECs. (B) Representative current traces after application of apamin in hiPSC-ECs. (C) Apamin sensitive currents ($I_{SK1\text{-}3}$) in hiPSC-ECs. (D) Representative current traces in the absence of apamin (control) in HCMECs. (E) Representative current traces after application of apamin in HCMECs. (F) Apamin sensitive currents ($I_{SK1\text{-}3}$) in HCMECs. (G) The protocol for recording currents. The holding potential was -40 mV, test potentials ranged from -80 mV to +50 mV for 400 ms.

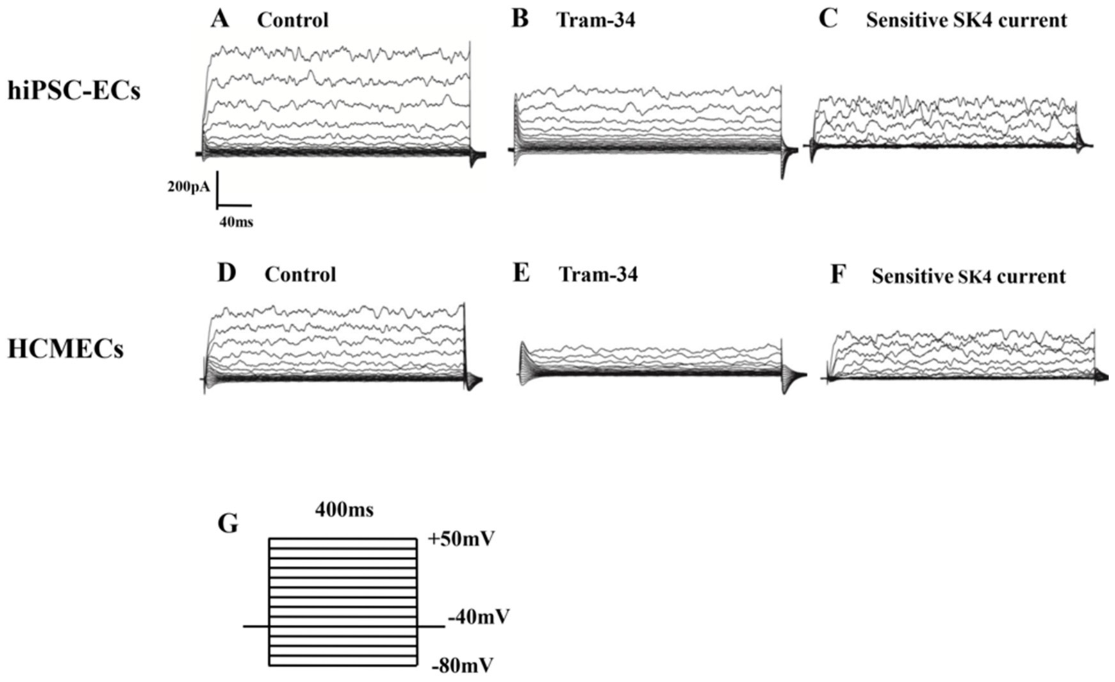


**Supplementary Figure S3. SK4 currents in hiPSC-ECs and HCMECs.** Membrane currents were recorded using the protocol shown in G. Tram-34 (1 μM), a specific blocker of SK4, was used to separate $I_{SK4}$ from other currents. (A) Representative current traces in the absence of Tram-34 (control) in hiPSC-ECs. (B) Representative current traces after application of Tram-34 in hiPSC-ECs. (C) Tram-34 sensitive currents ($I_{SK4}$) in hiPSC-ECs. (D) Representative current traces in the absence of Tram-34 (control) in HCMECs. (E) Representative current traces after application of Tram-34 in HCMECs. (F) Tram-34 sensitive currents ($I_{SK4}$) in HCMECs. (G) The protocol for recording currents. The holding potential was -40 mV, test potentials ranged from -80 mV to +50 mV for 400 ms.

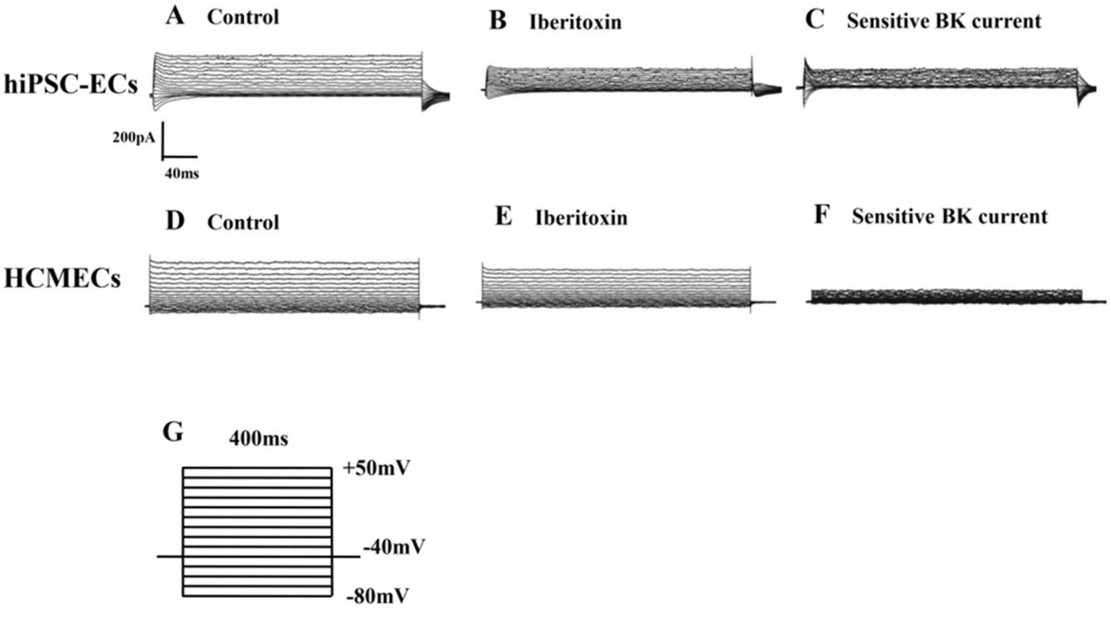


**Supplementary Figure S4. BK currents in hiPSC-ECs and HCMECs.** Membrane currents were

recorded using the protocol shown in G. Iberitoxin (100 nM), a specific blocker of BK current ($I_{BK}$), was used to separate $I_{BK}$ from other currents. (A) Representative current traces in the absence of iberitoxin (control) in hiPSC-ECs. (B) Representative current traces after application of iberitoxin in hiPSC-ECs. (C) Iberitoxin sensitive currents ($I_{BK}$) in hiPSC-ECs. (D) Representative current traces in the absence of iberitoxin (control) in HCMECs. (E) Representative current traces after application of iberitoxin in HCMECs. (F) Iberitoxin sensitive currents ($I_{BK}$) in HCMECs. (G) The protocol for recording currents. The holding potential was -40 mV, test potentials ranged from -80 mV to +50 mV for 400 ms.

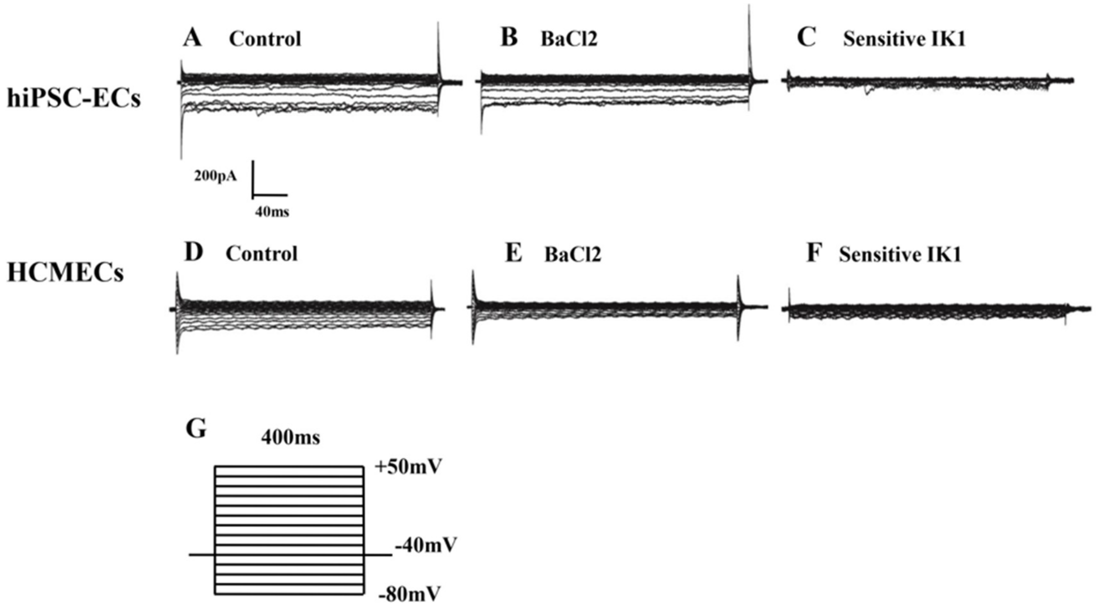


**Supplementary Figure S5. $I_{K1}$ in hiPSC-ECs and HCMECs.** Membrane currents were recorded using the protocol shown in G. $BaCl_2$ (100 μM), a blocker of $I_{K1}$ current, was used to separate $I_{K1}$ from other currents. (A) Representative current traces in the absence of $BaCl_2$ (control) in hiPSC-ECs. (B) Representative current traces after application of $BaCl_2$ in hiPSC-ECs. (C) $BaCl_2$ sensitive currents ($I_{K1}$) in hiPSC-ECs. (D) Representative current traces in the absence of $BaCl_2$ (control) in HCMECs. (E) Representative current traces after application of $BaCl_2$ in HCMECs. (F) $BaCl_2$ sensitive currents ($I_{K1}$) in HCMECs. (G). The protocol for current recording, with a holding potential of -40 mV and test potentials ranging from -120 mV to +50 mV for 400 ms.

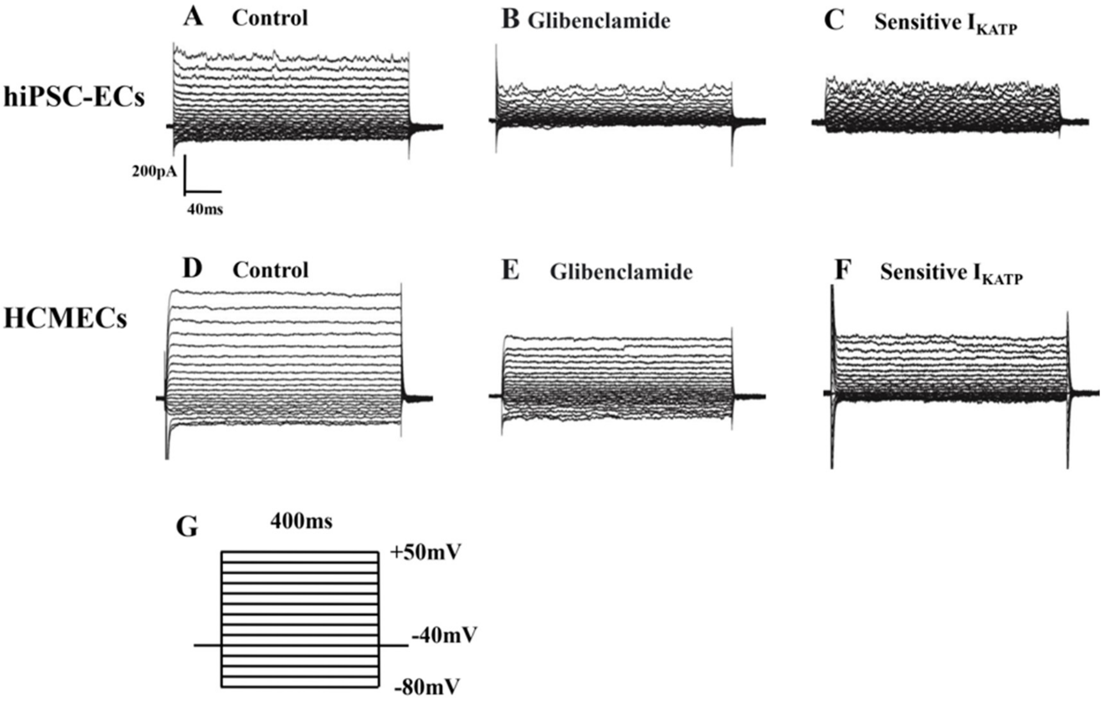


**Supplementary Figure S6. $I_{KATP}$ in hiPSC-ECs and HCMECs.** Membrane currents were recorded using the protocol shown in G. Glibenclamide (10 μM), a blocker of $I_{KATP}$ current, was used to separate $I_{KATP}$ from other currents. (A) Representative current traces in the absence of glibenclamide (control) in hiPSC-ECs. (B) Representative current traces after application of glibenclamide in hiPSC-ECs. (C) Glibenclamide sensitive currents ($I_{KATP}$) in hiPSC-ECs. (D) Representative current traces in the absence of glibenclamide (control) in HCMECs. (E) Representative current traces after application of glibenclamide in HCMECs. (F) Glibenclamide sensitive currents ($I_{KATP}$) in HCMECs. (G). The protocol for current recording, with a holding potential of -40 mV and test potentials ranging from -120 mV to +50 mV for 400 ms.
